# Isolated Percutaneous Endoscopic Gastrostomy Site Metastasis From Hypopharyngeal Squamous Cell Carcinoma

**DOI:** 10.7759/cureus.29123

**Published:** 2022-09-13

**Authors:** Nicholas D Luke, Penser Cardenas, Chad Phillip, Raji Mohammad, Ali Raza

**Affiliations:** 1 Medicine, St. George's University, True Blue, GRD; 2 Surgery, New York City Health and Hospitals Corporation (NYCHHC), Bronx, USA; 3 Pathology, New York City Health and Hospitals Corporation (NYCHHC), Bronx, USA

**Keywords:** surgical resection, chemotherapy, percutaneous endoscopic gastrostomy, hypopharyngeal carcinoma, metastatic disease

## Abstract

Head and Neck Squamous Cell Carcinoma (HNSCC) is a relatively uncommon malignancy due to the human papillomavirus or environmental factors such as excessive alcohol or tobacco use. Its most common metastatic locations are the lungs, bone, and liver. We are reporting a much more exceedingly rare site, a percutaneous endoscopic gastrostomy (PEG) site. HNSCC metastases and recurrences are commonly seen; however, they present complex challenges to manage successfully. Our presenting patient had an initial diagnosis of hypopharyngeal squamous cell carcinoma and then developed an isolated metachronous metastatic tumor at the site of his gastrostomy tract approximately one year later.

## Introduction

Head and Neck Squamous Cell Carcinoma (HNSCC) is the sixth most common cancer worldwide and arises on mucosal surfaces on the head and neck. It can present anywhere from the oral cavity to the hypopharynx and may cause atypical bleeding via ulceration or difficulty swallowing [1]. HNSCC can metastasize to lymph nodes and distant organs hematogenous. The malignancy spreads to distant metastatic sites such as the mediastinal and abdominal lymph nodes, the lungs, the liver, the bones, and very rarely to implantable medical equipment [2]. HNSCC can metastasize due to its ability to downregulate E-cadherin / catenin and pass through the epithelium thereafter [3]. Without the loss of E-cadherin / catenin integrity, HNSCC cannot advance and metastasize to other parts of the body. Our presenting patient displayed distant metastatic spread to a PEG tube tract used for nutritional intake when oral feeding was not viable (our patient had difficulty swallowing due to the HNSCC). The metastatic spread occurred about one year after the initial diagnosis of a hypopharyngeal HNSCC, and there was no detection of spread to other neighboring organs. This isolated event is sporadic, with a limited number of other case reports describing this phenomenon. Huang et al. and Mincheff describe how awareness of this tumor behavior and careful monitoring of the PEG tube can facilitate early detection of isolated metastatic spread, especially when using the pull-through method for gastrostomy tube placement [4,5]. This is crucial because the overall mortality after isolated PEG site metastasis was 87.1%, with a one-year survival rate of 35.5% [4].

## Case presentation

Our presenting patient is a 45-year-old male that presented initially with left anterior neck trauma after an assault. He was intoxicated, and he denied loss of consciousness and head trauma. A review of systems was positive for anterior neck pain, difficulty swallowing and spitting up blood. The patient had no difficulty breathing. On the physical exam, there was no pharyngeal erythema or blood in the pharynx, the uvula was midline, phonation was normal, and no stridor was present. Vital signs were within normal limits, and the airway was protected. An investigation of laboratory results is depicted below in Table 1.

**Table 1:** Pertinent positive laboratory findings seen in our patient upon presentation to the emergency room with reference ranges.

Pertinent Positive Laboratory Value	Laboratory Results	Reference Ranges
Sodium	128 mmol/L	135 - 145 mmol/L
Chloride	92 mmol/L	96 - 106 mmol/L
Creatinine	0.47 mg/dL	0.7 - 1.3 mg/dL
Calcium	8.1 mg/dL	8.5 - 10.2 mg/dL
Ethanol	351.2 mg/dL	0 - 0.02 mg/dL
White Blood Cell Count	4.02 x 10^3/mcL	4.5 x 10^3 - 11 x 10^3 / mcL
Hematocrit	30.9%	41% - 50% (Males)
Hemoglobin	10.8 g/dL	13.8 - 17.2 g/dL

**Figure 1:**
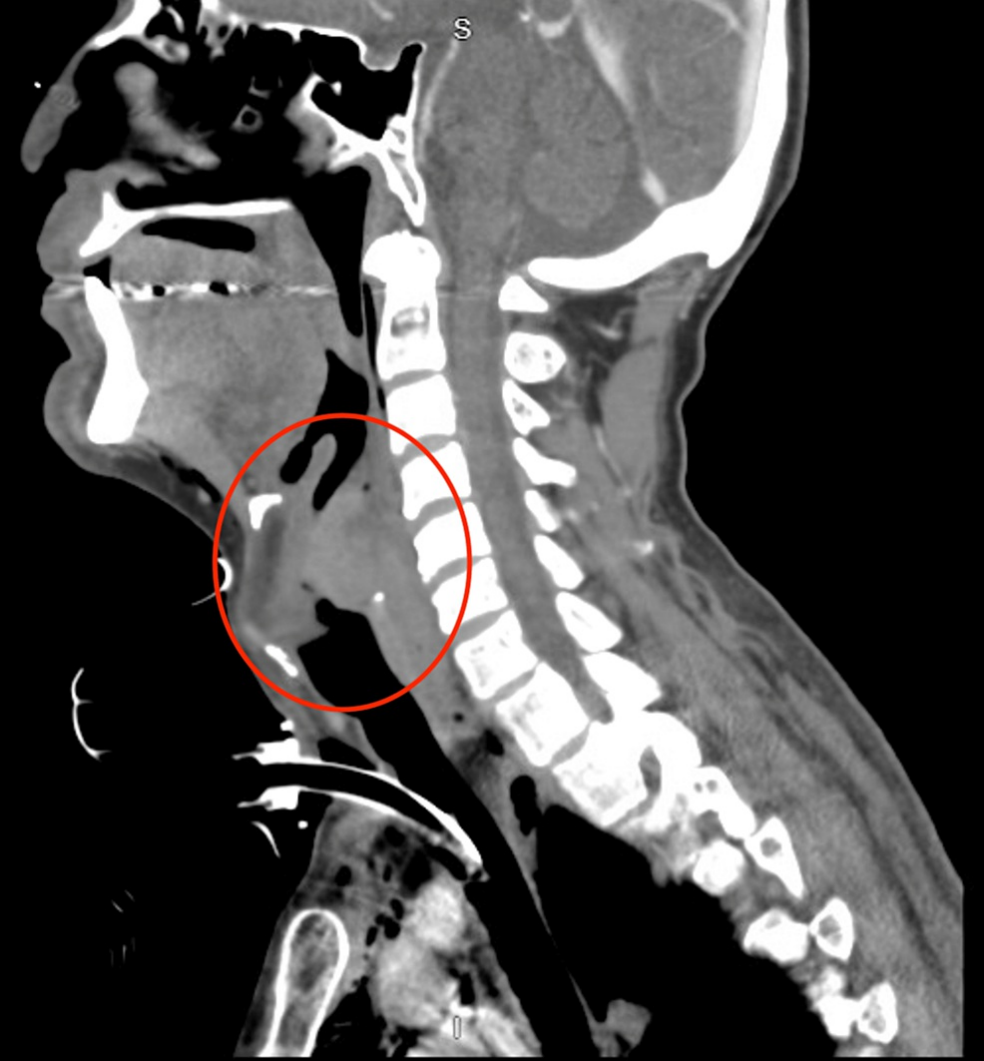
Soft tissue computed tomography found a large partially necrotic hypopharyngeal mass (red circle) measuring 4.8x4.4x3.3cm. Possibly originating from either retropharyngeal space or posterior mucosal space with involvement of aryepiglottic folds.

Further history revealed that the patient had been a smoker and alcoholic since 15. The patient endorses loss of appetite due to inability to tolerate solid food and weight loss greater than 30 pounds within a few months. Soft tissue computed tomography of the neck depicted a large hypopharyngeal mass with lymphadenopathy (a 3cm enlarged right cervical node and 2.5cm left cervical node) and no vascular injury or stenosis (Figure 1). Flexible fiberoptic laryngoscopy visualized a hypopharyngeal mass with bloody secretions obscuring the glottis's full view. Given concern for impending airway obstruction and a reported one month of dysphagia, the patient underwent tracheostomy and PEG creation.

**Figure 2:**
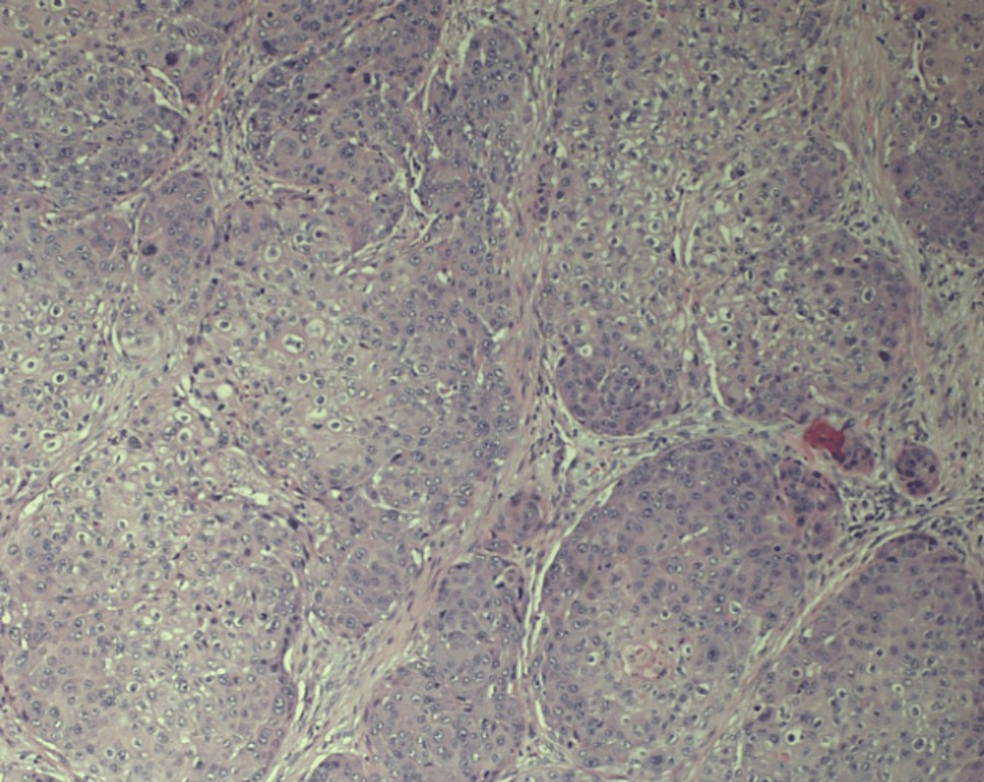
Biopsy of hypopharyngeal squamous cell mass with moderate to poor differentiation (Hematoxylin & Eosin stain, original magnification: x100). The size of the mass was 3x3.7x3.4cm.

The mass was biopsied at the time of tracheostomy and PEG creation (PEG was made during the initial presentation to the emergency department) via direct laryngoscopy and showed squamous cell carcinoma with moderate to poor differentiation (Figure 2). The mass was staged as CT2N2M0 stage 4a (Clinical Tumor-Node-Metastasis) at the primary tumor diagnosis time. The patient underwent concurrent chemoradiotherapy with cisplatin for three months. A subsequent computed and positron emission tomography scan showed a complete response two months after concluding chemoradiotherapy sessions. One year after the initial diagnosis, the patient presented with constipation and abdominal tenderness around the gastrostomy site (the tube was removed eight months after initial placement).

**Figure 3:**
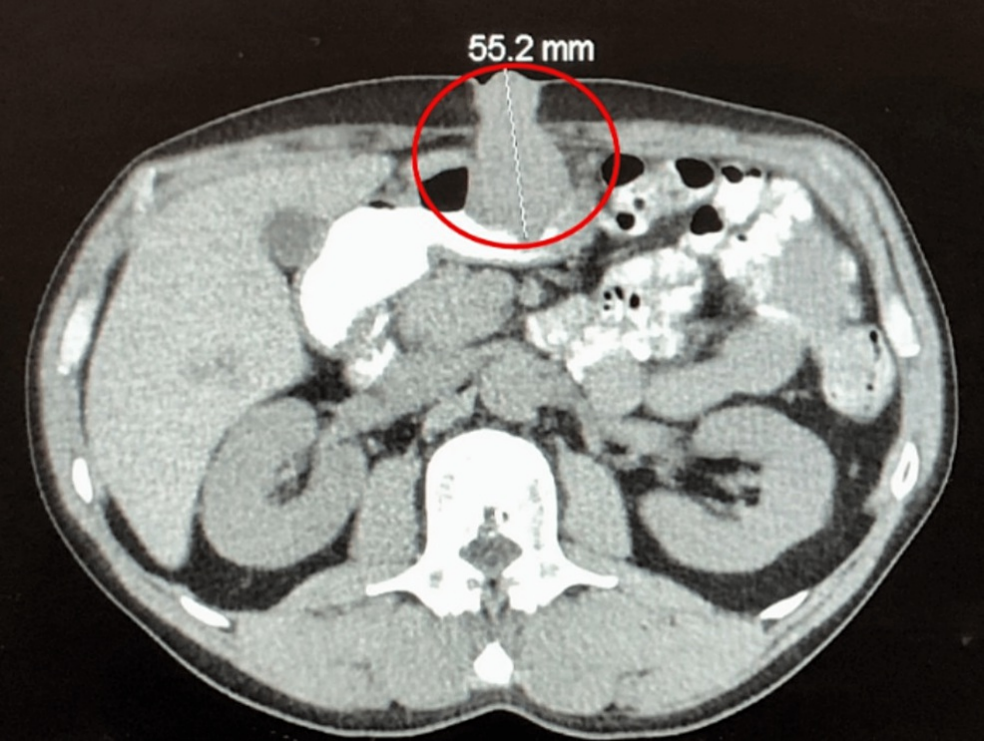
Computed tomography of the abdomen depicted a soft density (red circle) suggestive of a 5cm desmoid-like tumor at the percutaneous endoscopic gastrostomy site that extends onto the distal anterior surface of the antrum. No evidence of obstruction, free fluid, or pneumoperitoneum, and the appendix was unremarkable.

**Figure 4:**
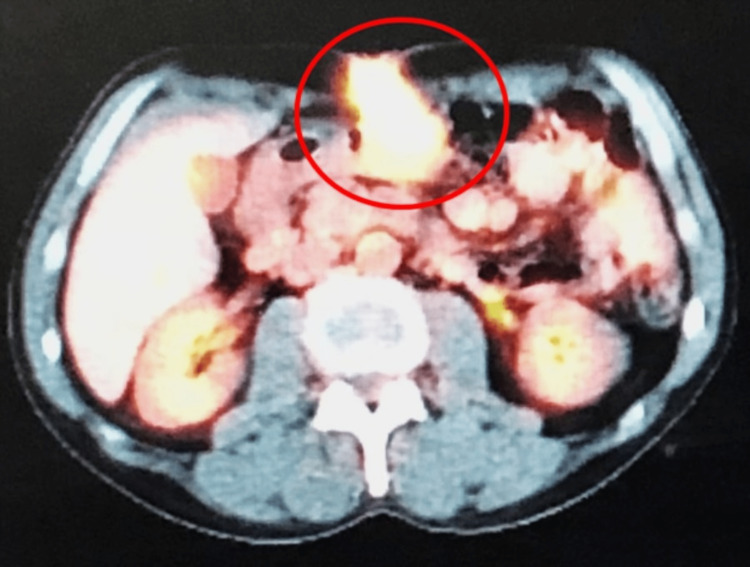
Positron emission tomography scan illustrated intense metabolic uptake localized to a soft tissue along the gastrostomy tract (red circle). Isolated recurrence at the percutaneous endoscopic gastrostomy tube tract extended below the fascia and at least onto the anterior aspect of the distal stomach.

The mass was biopsied during tracheostomy and PEG creation via direct laryngoscopy and showed squamous cell carcinoma with moderate to poor differentiation (Figure 2). The patient completed chemotherapy five months after the initial diagnosis. One year after the initial diagnosis, the patient presented with constipation and abdominal tenderness around the gastrostomy site (the tube was removed eight months after initial placement). There was a 1cm point of induration at the former PEG tube site, with no bleeding or discharge. A computed tomography scan of the abdomen showed a soft density suggestive of a desmoid-like tumor at the PEG site that extends onto the distal anterior surface of the antrum (Figure 3). Computed tomography of the chest showed no lung metastasis and no lymphadenopathy. Biopsy of the abdominal mass depicted squamous cell carcinoma. A positron emission tomography (PET) scan showed isolated recurrence at the PEG tube extending below the fascia and at least onto the anterior aspect of the distal stomach (Figure 4). Following these findings, the patient underwent en-bloc resection of the abdominal wall tumor with distal gastrectomy. Final pathology revealed a 5cm tumor involving the anterior gastric wall and gastrostomy stoma with negative margins. The report specifically mentions that the morphology is consistent with metastatic hypopharyngeal carcinoma. Freezing sectioning was negative for proximal gastric margins. The estimated blood loss was 30 ml, and there were no intraoperative complications. 

Postoperatively, the patient had incisional pain controlled with hydromorphone pushes and no nausea or vomiting. The patient had episodes of hypertension treated with labetalol on several postoperative days. He was kept on nasogastric feeding for five days postoperatively. The patient was afebrile, ambulating, and passing flatus, and the incision was clean and intact throughout the postoperative hospital stay. A palliative chemotherapy regimen with paclitaxel, pembrolizumab, and carboplatin was initiated. 

**Figure 5:**
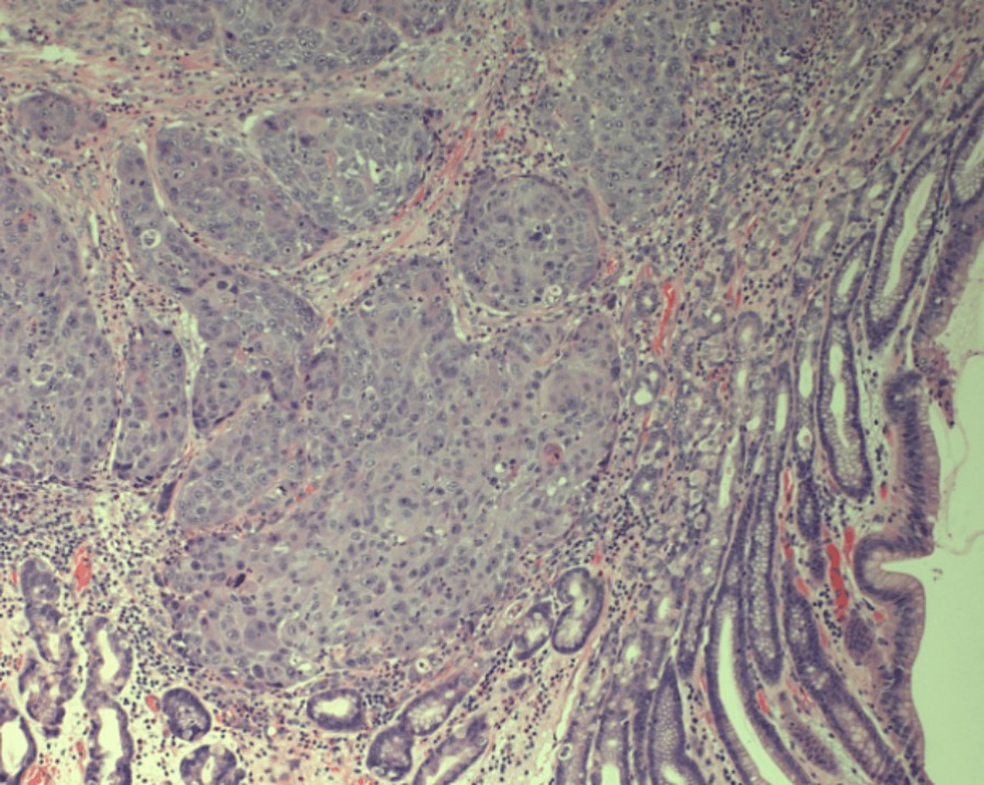
Squamous cell carcinoma of similar morphology to hypopharyngeal carcinoma involving the gastric wall and mucosa (Hematoxylin & Eosin stain, original magnification: x100).

**Figure 6:**
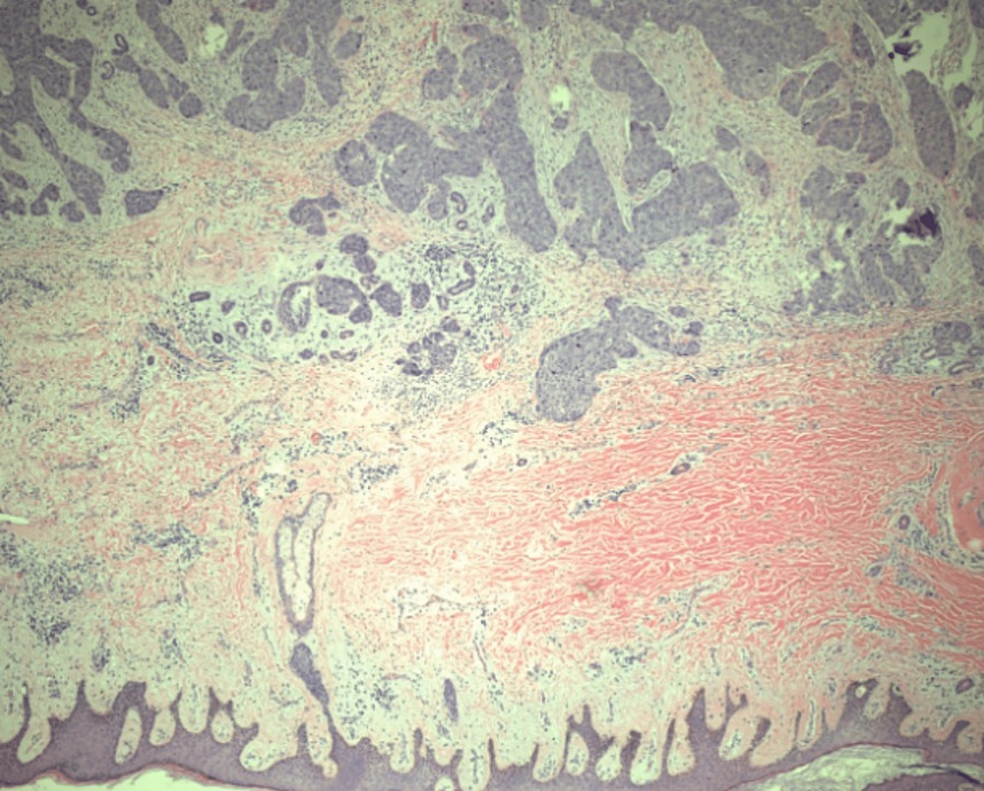
Skin component of percutaneous endoscopic gastrostomy with metastatic squamous cell carcinoma (Hematoxylin & Eosin stain, original magnification: x40).

Surgical pathology of the head and neck biopsy showed invasive squamous cell carcinoma with moderate differentiation. The pathology report of the abdominal biopsy showed similar squamous cell carcinoma morphology to that of the hypopharyngeal mass with moderate to poor differentiation (Figure 5, 6). The metastatic site involved the gastric wall, including the mucosa (Figure 5). A skin component of the percutaneous endoscopic gastrostomy depicted squamous cell carcinoma as well (Figure 6). The abdominal mass measured 5cm. Lymphovascular invasion was present, along with the invasion of the gastric mucosa, submucosa, muscularis propria, soft tissue, subcutaneous tissue, and the skin's dermis. Necrosis was present.

## Discussion

Isolated metastatic spread of HNSCC to the PEG tube site is an infrequent occurrence that was first documented in 1989 [6]. As of 2022, there are only 83 reported cases. Over time, PEG tubes have continuously proven to be helpful when oral nutritional intake is not feasible. They are typically more tolerable and user-friendly than nasogastric tubes and may have a lower risk of aspiration pneumonia [7]. These benefits outweigh the incidence of complications associated with them. However, special attention is required when utilizing a PEG tube in a patient with HNSCC due to potentially high one-year mortality rates if metastatic HNSCC does occur [4]. Although there is not much in the way of epidemiological data, Zhang estimates that the incidence of PEG site metastasis is about 0.5% to 1%, based on fewer than 50 cases reported up until 2014 [8].

Another case report examined three patients presenting similar to ours and noted that the survival time after PEG site metastasis was poor [9]. Metkus, Cognetti, and Curry published survival times in months for their patients: 20.6, 13.37, and the third patient did not have a survival time or cause of death available [9]. The cause of death for the two other reported patients included disease metastasis and complication of treatment of local recurrence [9]. All three patients had the 'pull-through' method for PEG placement, and metastatic disease was noted about 6-9 months after placement [9]. The pull-through method has the highest incidence of gastrostomy site metastasis, presumably due to cancer seeding as the gastrostomy tube passes through the pharynx twice [9]. This method is the only technique that requires the gastrostomy tube to pass through the pharynx twice. The seeding theory is supported by one study, which demonstrated that 9 of 40 patients with head and neck cancer who underwent PEG creation had tumor cells at the stomal site immediately at the procedure's end. 

Our patient presented with a more delayed onset of metastasis after almost a year with vague symptoms (abdominal tenderness, constipation), and the pull-through method was also used. Many case reports on this phenomenon share common ground in which the pull-through method of PEG placement was used. This common denominator should push clinicians to avoid the pull-through method in future cases and rely on other insertion methods. Besides the pull-through method, clinicians can use the guide wire, the' push' method, or the introducer (Russell) method [10]. The introducer method was associated with the smallest incidence of gastrostomy site metastasis in one small single-institution series [9], likely due to avoiding contact with the pharynx. 

Treatment of metastatic HNSCC usually involves chemotherapy. If the tumor is positive for PD-L1 (Programmed death-ligand 1), pembrolizumab can be incorporated into the treatment plan [11]. However, based on retrospective data, oligometastatic and metachronous tumors (like our presenting patient) are more likely to benefit from an aggressive ablative approach [11]. Such evidence is based on trials like the randomized phase II SABR-COMET trial, which included 99 patients with one to five metastases [11]. The trial demonstrated that patients that underwent local ablation had improved survival rates (significantly improved 5-year overall survival with local ablation (42% vs. 17%, p = .006)) [11]. It should be noted that this data is based on a small number of retrospective studies and a highly selected group of patients [11]. 

HNSCC is a challenging malignancy to combat. Even after treatment of the primary tumor, about 50% of patients will experience a recurrence of the disease [12]. Once recurrence occurs, the prognosis is poor, with a median survival of about 12 months even when reinstating a treatment plan [12,13]. If the tumor recurs, the clinician should educate the patient that a cure is typically not a realistic option. Controlling the patient's symptoms should take priority, especially since there is little evidence that salvage surgery or re-irradiation will be significantly helpful in improving patient survival [12]. Elbers et al. performed salvage surgery on 189 patients diagnosed with recurrent HNSCC [14]. Elbers et al. discovered that larynx carcinomas had a more favorable prognosis than oro/hypopharyngeal carcinomas [14]. They also noted that the five-year overall survival rate with the surgical intervention was 33%, and the median overall survival was 18 months [14]. Paleri and Kelly examined whether re-irradiation therapy may be useful in patients with recurrent disease and when surgical resection is not feasible [15]. Via a decision analysis model, they found that re-irradiation had a superior quality-adjusted life year (QALY) score in comparison to palliative care in select patients (20 weeks versus 15 weeks for palliation) [15]. Overall, clinicians should guide the patient's management of recurrent disease based on circumstance. If the clinician is intent on curing the patient, salvage surgery has typically proven to be the better choice, assuming the patient has a resectable tumor and is in good health [12]. 

Furthermore, this case is unique in that there appears to be only one other reported case of metastasectomy for control of the disease [16]-Fonseca et al. report an abdominal wall resection with total gastrectomy following their patient's initial neck surgery.

## Conclusions

Metastatic HNSCC to PEG tube site tracts is a sporadic occurrence. The metastatic site can be treated with surgery or re-irradiation, but the prognosis is typically poor. When clinicians plan to utilize the pull-through method to implant PEG tubes on patients with HNSCC, they should maintain a high index of suspicion of tumor recurrence/metastases to the PEG site. This tumor behavior is atypical and poses a severe challenge when managing patients with HNSCC.
